# Complete mitogenome for the ber fruit fly, *Carpomya vesuviana* (Diptera: Tephritidae) from Toksun, Northwest China and its phylogenetic relationship within family Tephritidae

**DOI:** 10.1080/23802359.2019.1682475

**Published:** 2019-11-05

**Authors:** Ping-Fan Jia, Jian-Hong Liu, Jin Xu, Xiao-Jun Li, Wen-Li Dan

**Affiliations:** a Key Laboratory of Forest Disaster Warning and Control of Yunnan Province, Southwest Forestry University, Kunming, China;; b School of Nursury and Rehabillitation, Xinyu University, Xinyu, China

**Keywords:** *Carpomya vesuviana*, mitochondrial genome, molecular phylogeny

## Abstract

*Carpomya vesuviana* (Diptera: Tephritidae), commonly known as the ber fruit fly, is the most destructive insect pest of 'ber' (jujube) throughout Asia and Western Europe. Complete sequence of the mitogenome of *C. vesuviana* has been determined in this study. The circular genome is 15,267 bp long and contains a standard gene complement, that is, the large and small ribosomal RNA subunits, 22 transfer RNA genes, 13 genes encoding mitochondrial proteins, and a non-coding A + T-rich control region. The phylogeny showed that *C. vesuviana* of subfamily Trypetinae was monophyletic and clearly separated from both Dacinae and Tephritinae with maximum support (*p* = 1).

*Carpomya vesuviana*, commonly known as the ber fruit fly, is the most destructive insect pest of ‘ber’ (jujube) throughout Asia and Western Europe (Azam et al. 2004). The fly is oligophagous and has been recorded on hosts from *Ziziphus spp.* fruits (Norrbom 1997). At present, there is only one mitochondrial genome data in subfamily Trypetinae of Family Tephritidae. In this study, we reported the complete mitogenome of *C. vesuviana* in subfamily Trypetinae. The results would greatly facilitate phylogenetics, population genetics, and species identification.

The adult male flies were caught from Toksun (42.27°N, 88.09°E), Northwest China, on 13 August 2018. Specimen was deposited in the museum of Southwest Forestry University (Voucher Xinjiang 20180813), Kunming, China. Genomic DNA was extracted from adult fly following the manufacturer’s instruction in the DNeasy Blood and Tissue kit (Qiagen, Hilden, Germany) and then sequenced and assembled using Illumina’s HiSeq2000 platform (Illumina, San Diego, CA). The sequence was preliminarily aligned within the CLUSTAL X programme in BioEdit software. Protein-coding genes (PCGs), rRNA genes were predicted by using MITOS tools (Bernt et al. 2013), and tRNA was done through tRNAscan-SE (Lowe and Chan 2016).

The complete mitogenome of *C. vesuviana* is 15,267 bp long in size (GenBank MN095236). The base-pair composition is 39.82% for A, 13.76% for C, 9.88% for G, and 36.54% for T. The mitogenome contains 13 PCGs, 22 transfer RNA genes, 2 ribosomal RNA genes, and a major non-coding region known as the CR (control region). J-strand codes nine PCGs (*NAD2-3*, *NAD6, COX1-3, CYTB, ATP6*, and *ATP8*) and 14 tRNAs, while N-strand codes four PCGs (*NAD1, NAD4, NAD4l*, and *NAD5*), eight tRNAs, and two rRNAs (16S rRNA and 12S rRNA). The gene arrangement of mitogenome is identical to the most common type of the putative ancestor of insects (Boore 1999; Cameron 2014).

To validate the phylogenetic position, the mitogenomes of 20 species in family Tephritidae and the two outgroups, *Drosophila suzukii* and *Drosophila melanogaster* (Diptera: Drosophilidae) were clustered together to construct Bayesian tree by using the BI method in MrBayes version 3.2.6 (Huelsenbeck and Ronquist 2001; Dereeper et al. 2010, 2008). The result indicated that *C. vesuviana* in subfamily Trypetinae was monophyletic and clearly separated from both Dacinae and Tephritinae. In subfamily Dacinae, genus *Dacus* and *Zeugodacus* formed a sister clade to genus *Bactrocera*. The topology of the BI tree had maximum support (*p* = 1) (Figure 1). Furthermore, three subfamilies were correctly identified as assigned and monophyletic Dacinae, Trypetinae and Tephritinae (Figure 1). In conclusion, the mitochondrial genome of *C. vesuviana* reduced in the present study can provide essential DNA molecular data for further phylogenetic and evolutionary analysis.

**Figure 1.**
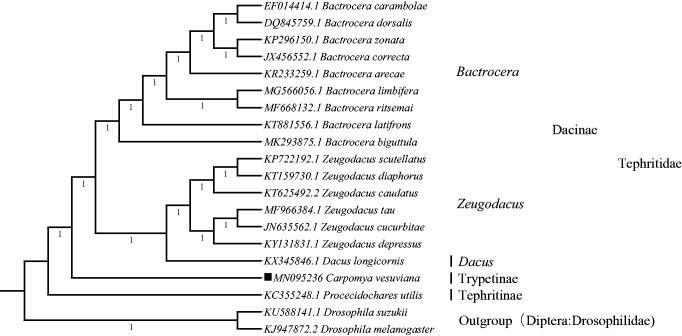
Molecular phylogeny for *Carpomya vesuviana* and the related 17 species in family Tephritidae and two outgroups based on complete mitogenome. Tree was constructed by Bayesian Inference (BI) method. Genbank accession numbers lie before the scientific name of species. The position of *C. vesuviana* is marked with solid square shape.
